# Risk factors for Catheter-Associated Urinary Tract Infection (CAUTI) in sepsis patients at RSPAD GATOT SOEBROTO 2022: a quantitative study

**DOI:** 10.1017/ash.2025.148

**Published:** 2025-09-03

**Authors:** Theresia Leonita, Soroy Lardo, Maria Selvester Thadeus, Marlina Dewi Astuti, Martaviani Budiastuti, Jonny

**Affiliations:** 1 Faculty of MedicineUniversitas Pembangunan Nasional “Veteran” Jakarta, Jakarta, Indonesia; 2 Division of Tropical and Infectious Diseases, Department of Internal MedicineGatot Soebroto Army Hospital, Jakarta; 3 Department of Pathology AnatomyUniversitas Pembangunan Nasional “Veteran” Jakarta, Jakarta, Indonesia; 4 Department of Internal MedicineUniversitas Pembangunan Nasional “Veteran” Jakarta, Jakarta, Indonesia; 5 Head of PPI CommitteeGatot Soebroto Army Hospital, Jakarta; 6 Division of Nephrology & Hypertension, Department of Internal MedicineGatot Soebroto Army Hospital, Jakarta

**Keywords:** UTI, CAUTI, sepsis patient, risk factors, Indonesia

## Abstract

**Background:** Urinary tract infection (UTI) is the most dominant case, around 40% of healthcare-associated infections (HAIs). UTI related to catheter placement called as Catheter-Associated Urinary Tract Infection (CAUTI). Catheterization is considered as a port of entry that lead to infection. In sepsis patients, CAUTI can significantly affect clinical outcomes. Prolonged CAUTI can worsen but can be prevented via suitable intervention, particularly in septic patients with urine catheters. To effectively prevent and manage diseases, gathering data focusing on surveillance is essential. Hence, examining multiple risk variables associated with CAUTI is vital, including age, gender, diabetes mellitus, kidney failure, frequency and duration of catheterization, and duration of antibiotic usage before urine culture. **Method:** A quantitative study using a cross-sectional design by selecting samples using total sampling was conducted at RSPAD Gatot Soebroto (n=42). All sepsis patients using catheters met the inclusion criteria. The data obtained was analysed (univariate, bivariate and multivariate), which will be presented in table and narrative format. **Results:** It was found that 21 sepsis patients with catheters confirmed CAUTI. Risk factors in septic patients with catheters that have a significant relationship with CAUTI are diabetes mellitus (p=0.013), kidney failure (p=0.005), length of stay (p=0.013), duration of antibiotic usage before urine culture (p=0.031), frequency of catheterization (p=0.028), and duration of catheterization (p=0.013). However, age (p=0.739) and gender (p=0.757) did not have a significant relationship. In the multivariate test was found that the most significant variables were kidney failure (p=0.006; OR=22.219; 95%CI=2.424- 293.744) and duration of catheterization (p=0.009; OR=19.147; 95%CI=2.070-177.149). **Conclusion** : Our findings indicate that kidney failure and duration of catheterization are the most significant risk factors for septic patient who develop CAUTIs. To enhance the clinical outcomes of sepsis patients prone to CAUTI, it is crucial to identify the risk factors as a part of treatment management and infection prevention control.

